# Positive Reinforcement (R+) Horse Training in Practice: Evaluation of Online Trailer-Training Demonstrations

**DOI:** 10.3390/ani16111667

**Published:** 2026-05-29

**Authors:** Helena G. Harris, Sue M. McDonnell

**Affiliations:** 1 Animal Welfare and Behavior Graduate Program, University of Pennsylvania School of Veterinary Medicine, Kennett Square, PA 19348, USA; hgharris07@gmail.com; 2 Havemeyer Equine Behavior Laboratory, Department of Clinical Studies, New Bolton Center, University of Pennsylvania School of Veterinary Medicine, Kennett Square, PA 19348, USA

**Keywords:** horse welfare, trailer-training, trailer-loading, positive reinforcement, operant conditioning, horse transportation

## Abstract

Operant conditioning that includes primarily positive reinforcement (R+) has been shown to be an effective and welfare-friendly method of training horses. Despite this, operant conditioning and R+ are not fully understood by many horse trainers who claim to advocate for its use. Anecdotal observations suggest that some professional trainers who claim to use R+ do not use R+ primarily, even in publicly offered educational demonstrations. To better understand readily available training materials regarding the use of R+ with horses, we reviewed a sample of 20 online trailer-training demonstrations to evaluate the extent to which R+ was used. Based on the percentage of trainer actions judged to be R+ versus negative reinforcement (R-), positive punishment (P+), or negative punishment (P-), eight of the 20 (40%) demonstrations included R+ exclusively. Three additional demonstrations included primarily R+ (> 90% of trainer actions), with the remainder R-. Seven used mostly R+ (55–80% R+ in combination with R-), and two used little R+ (16–23%), with the remainder mostly R-. The percentage of positive behavioral responses of the horse toward loading progress was strongly positively associated with the percentage of R+ trainer actions (r^2^ = 0.90, *p* < 0.0001). The results of this sample confirm that not all online demonstrations of R+ accurately portray primarily R+.

## Simple Summary

Positive reinforcement (R+) has been shown to be an effective and welfare-friendly strategy for training horses. Despite this, R+ is still not fully understood by many of the horse trainers and educators who claim to endorse its use. Our anecdotal observations indicate that some trainers claim to use and teach R+ methods, when in fact what they teach is not primarily R+. To better understand the nature of readily available educational materials regarding R+ training of horses, we reviewed a sample of 20 trailer-training demonstration videos presented online by individuals claiming to use R+ methods. Eight of the 20 trainers (40%) exclusively used R+. Three of the 20 trainers primarily used R+ (>90% of their actions). Seven used mostly R+ (55–82% of their actions), with the remaining actions judged to represent negative reinforcement (R-). The remaining two trainers (10%) used little R+ (<25% of their actions), with mostly negative reinforcement (R-) and positive punishment (P+). As we expected, among the various demonstrations, the greater the percentage of R+ trainer actions, the greater the percentage of positive horse behavioral responses. The results of this sample confirm that not all online demonstrations of R+ accurately portray primarily R+.

## 1. Introduction

The training of a horse involves a high degree of human interaction, often for years or decades. Ensuring that trainers and equine professionals are employing and teaching humane, scientifically sound training practices is a key component of equine welfare. Operant conditioning that includes exclusively, or at least primarily, positive reinforcement (R+) has been shown to be an effective and welfare-friendly strategy for training horses and other animals [1,2].

As background, in the early 1900s behavioral scientists sought to understand the “laws of learning”. B. F. Skinner pioneered systematic investigation into how consequences influence behavior. He named this consequence-based behavior modification *operant conditioning*, based on the concept that “organisms operate on their environment to produce consequences”. Resulting from this early work, in learning science, consequences are classified according to whether they increase or decrease the frequency and strength of the behavior they follow. *Reinforcement* refers to consequences that increase the behavior. The two distinct forms of reinforcement include (1) the occurrence of a positive, desired stimulus or condition following a behavior, called *positive reinforcement*, and (2) removal of a negative (aversive or undesirable) stimulus or condition following a behavior, called *negative reinforcement* (R-). *Punishment* refers to consequences that decrease the frequency or strength of a behavior, also with two distinct forms. These include (1) occurrence of an aversive stimulus following a behavior, called *positive punishment* (P+), and (2) withholding of a positive stimulus following a behavior, called *negative punishment* (P-). These four possible consequences influencing behavior are often explained using a 2 × 2 table. So, in contemporary behavior modification practice, R+, R-, P+, and P- are commonly referred to as *the four quadrants* of operant conditioning ([3], for current review of).

It has been our experience that numerous horse training clinics and online demonstrations meant to educate the public about the application of operant conditioning methods often appear to misunderstand the use of R+. For example, we commonly encounter horse professionals who describe themselves as using primarily or exclusively R+, but whose methods include little use of R+. We have also observed such trainers demonstrating and promoting the use of primarily R- and P+, including the use of flooding, a method that leverages emotional distress to shape behavior by subjecting the horse to a prolonged, stressful stimulus [4,5]. Contrary to some practitioners’ claims, these methods tend to increase the risk of physical and emotional harm to horses, and compromise the safety of human handlers [4]. Many popular equine practitioners and educators also inaccurately describe and interpret equine behavior, emotional affect, and cognition as validation for their training methods [5,6].

Although we are unaware of any current data on how equestrians access educational materials related to horse training, in our daily work with horse owners, riders, and professionals of varying backgrounds, it is our impression that a common resource is online horse training videos. Readily available online videos range from professionally produced demonstrations designed to promote a commercially marketed training program, to casually recorded sessions posted by amateur equestrians. To critically assess the scope of readily available educational materials regarding the use of R+ to train horses, the main objective of our work reported here was to review a sample of online demonstrations presented by purported “positive reinforcement trainers” to evaluate the extent to which R+ was used. We chose to focus on demonstrations of training horses to load onto a transport vehicle, commonly known in North America as “trailer-training.” Various techniques for R+ training of horses and other livestock for transport loading have been described [7,8,9]. In brief, the goal is to positively reinforce naturally expressed exploratory behaviors to shape voluntary entry into a transport vehicle. Positive reinforcement training often includes pre-conditioning to approach and/or follow a handler or an object. One such technique is known as “target training”, which involves training the animal to approach and/or follow a target object which is reinforced with food or positive tactile stimulation. Such pre-training typically includes a secondary reinforcer, sometimes referred to by trainers as a “bridge” or “marker”. The secondary reinforcer used is usually an audible sound such as a clicker or handler utterance. Accordingly, this is commonly known as “target/clicker” or “clicker” training [8,10]. An additional goal of R+ trailer-training may be what is called “self-loading”, where the horse voluntarily walks onto the transport vehicle when it is presented (without being led by a handler). Some trainers work without a halter and lead on the horse, known as “at liberty” training. In this scenario, the horse is loose within a perimeter fence, but otherwise free to move unrestrained.

## 2. Materials and Methods

### 2.1. General Approach

For each of the 20 online trailer-training demonstrations presented as positive reinforcement training, we judged each trainer action/interaction (TA) as representing R+, R-, P+, or P-. We also recorded the corresponding behavioral responses of the horse as positive, negative, or neutral in terms of reaching the criterion of walking voluntarily into the trailer.

### 2.2. Sample of Online Demonstrations

Using Google’s public search engine, we ran queries using the terms *positive reinforcement*, *clicker training*, *R+*, and *positive training*, with *trailer training*, *trailer loading*, and *horse trailer*. The searches were conducted between 20 July and 5 October 2025. Of the 35 initial search results, we selected a sample of 20 videos published between 2011 and 2025 that met the following criteria: (a) the video was published to a digital platform such as YouTube, TikTok, Facebook, Instagram, or individually hosted websites, (b) the video title, description, or audio narration of the demonstration included the phrase *positive reinforcement*, *clicker training*, or *R+*, (c) the video included a horse, donkey, or mule being trained to walk into a horse trailer (either ramp or step-up entry), (d) the camera view adequately captured the handler’s actions/interactions, as well as the horse’s head (with ears visible), face, body, tail, legs, and feet, (e) the video included at least 1 min of actual handler–animal interaction, and (f) the video was published at normal speed (1×) vs. fast motion.

### 2.3. Trainer’s Actions with Horse

Table 1 lists specific trainer actions/interactions (TAs) common among various methods of training horses to load for transport, with the corresponding form of operant reinforcement (R+ or R-) or punishment (P+ or P-). For each 15 s interval throughout the demonstration, we recorded the TAs. These evaluations were conducted by a single investigator (HGH, an experienced equine behavior clinician with academic training in operant conditioning science) in consultation with another, similarly trained and experienced equine behavior specialist (SMM).

### 2.4. Horse Behavioral Responses

Table 2 defines and illustrates 36 behaviors commonly seen in the trailer loading context and that were observed in this sample of demonstrations. Each of these are categorized as reflecting positive, negative, or neutral progress toward the criterion of voluntarily walking into the trailer. For each 15 s interval, we recorded the behavioral responses of the horse to each corresponding trainer action.

### 2.5. Additional Observations Recorded

We noted the following additional conditions: whether the horse was fitted with a halter and lead rope or not (known as “at liberty”), the configuration of the entrance into the trailer (ramp or step-up), whether or not the horse had previous loading (or training exposure), whether the horse was naïve to the particular transport vehicle, the sex of the horse, the weather conditions (warm or cold), whether the trainer was professional or amateur, and the date of video publication.

### 2.6. Analysis

For each demonstration, we calculated the percentage of the total TAs that were judged to represent R+. We similarly calculated the percentage of the total behavioral responses of the horse that were judged to be positive (vs. negative or neutral) in terms of voluntarily walking into the trailer. Pearson’s R was used to evaluate the association between the percentage of R+ TAs and the percentage of positive behavioral responses of the horse. Fisher’s exact test was used to compare the proportions of “at liberty” versus use of halter and lead among trainers categorized as using *Primarily* R+ vs. *Mostly* or *Little* R+. Statistical tests were done using Statistix 10, Analytical Software, Tallahassee, FL 32312, USA.

## 3. Results

### 3.1. Resulting Sample

The resulting sample of 20 trailer-training video demonstrations ranged in length from 1 to 31 min, with the actual duration of recorded trainer–animal interactions ranging from 1 to 7 min. The trainers included 19 professional trainers and one amateur. All 20 video demonstrations involved horses (no videos involving mules or donkeys had been identified in the entire search). The horses included six mares, 13 geldings, and one stallion. Six horses were naïve, and the remaining 14 experienced at loading for transport or had had previous training sessions. Seven of the horses were fitted with a halter and lead, while 13 were at liberty. Sixteen of the trailers featured a ramp entrance, and the remaining four trailers were step-up.

### 3.2. Percentage of R+ TAs

For eight of the 20 (40%) trailer-training demonstrations in this sample, all the TAs were judged to represent R+ *Exclusively*. For an additional three of the demonstrations, more than 90% of the TAs were judged to represent R+. Therefore, we categorized these 11 (55%), with greater than 90% of TAs representing R+, as *Primarily R+*. For seven of the 20 (35%) demonstrations, 55–82% of the TAs were judged to represent R+, with the remaining actions representing R-. We categorized these seven as *Mostly R+*. For the remaining two demonstrations, only 16% and 23% of the TAs were judged to represent R+. Accordingly, we categorized these two as demonstrating *Little R+*. In terms of operant conditioning, these two included mostly R- (75% and 78%).

### 3.3. Association of R+ and Horse Response

There was a very strong positive association of the percentage of R+ TAs and the percentage of horse behavioral responses judged to be positive (Pearson’s r = 0.9483, 18 df, *p* < 0.0001).

### 3.4. Association of R+ with “At Liberty” Handling

Of the 11 demonstrations categorized as *Exclusively* or *Primarily R+*, 10 were “at liberty” and one was with halter and lead. In contrast, of the nine remaining demonstrations, three were at liberty and six were with halter and lead. The difference is highly significant (Fisher’s exact test, *p* < 0.05).

### 3.5. Other Data Recorded

We judged that there were too few samples to explore potential associations with ramp vs. step-up entrance to the trailer, naïve vs. experienced with the particular transport vehicle, sex, weather, or professional vs. amateur trainer.

## 4. Discussion

To estimate how accurately R+ training of horses is represented in educational materials available to equestrians, we examined a sample of online trailer-training video demonstrations of R+ to evaluate whether they relied solely on R+, and if not, what additional methods were used. In our sample of 20 such video demonstrations, 40% included solely R+ trainer actions. An additional three videos that included greater than 90% R+ trainer actions brought the total to 60% of our sample that we called *Exclusively* or *Primarily* R+. A total of 35% of the videos included 55% to 82% R+ in combination with R-, which we called *Mostly* R+. Interestingly, 10% of the videos included less than 25% R+ trainer actions, which we called *Little* R+. These results support our anecdotal impression that not all horse trainers who demonstrate R+ online use only R+.

This study was not designed to test the effectiveness of R+. However, we shared the observation that in this sample of 20 video demonstrations, the percentage of positive behavioral response from the horses in terms of progress toward the goal of calmly entering the transport vehicle was significantly associated with the percentage of R+ actions of the trainer. Positive reinforcement has been shown to result in better training outcomes [11] and to lower stress levels in horses [1,9,12]. We find this particularly apparent when acclimating or systematically desensitizing horses and other animals to naturally aversive husbandry circumstances. Specific to rehabilitating horses for loading for transport, Ferguson and Rosales-Ruiz [9] found that conflict behaviors that had developed in horses previously trained with aversive methods disappeared completely when re-trained using R+ methods. Not only was there an extinction of the conflict behaviors, but the re-trained voluntary loading response also generalized to other transport vehicle configurations, as well as to other human handlers.

In this sample, the proportion of “at liberty” demonstrations that were judged to involve R+ exclusively or primarily was significantly greater than the proportion of demonstrations in which the horse was handled with a halter and lead. At least three related issues come to mind as potential contributors to this difference. Firstly, we speculate that only skilled R+ trainers are likely to offer a demonstration with the horse at liberty. That is because when a horse is at liberty, in other words, free to leave, consistent use of R+ is typically required to maintain the horse’s interest in entering a transport vehicle. Secondly, the use of R- and P+ can be counterproductive when working at liberty, as any intentional or unintentional aversive actions of the trainer tend to cause the horse to withdraw from the interaction. Finally, when training a horse without a halter and lead rope, the risk of inadvertently using aversive stimuli is greatly reduced.

Animal trainers may employ R+ in combination with R-, either deliberately or inadvertently. In equitation science, systematic use of R+ together with R- has been termed *combined reinforcement* [13]. It typically involves applying light pressure to elicit the desired response, such as a gentle nudge or mild threatening gesture in the desired direction or movement, followed immediately by R+. When executed skillfully, this combination can be both effective and humane. Some evidence suggests that, in certain contexts, it may enhance both learning efficiency and retention compared to the use of R+ or R- alone [14]. The aim is to guide or cue the horse using a mildly aversive or conditioned negative stimulus while remaining below the threshold that induces fear or constitutes punishment. Achieving this consistently requires highly refined skills, including rapid recognition of the animal’s affective state and precise, moment-to-moment adjustment of pressure. In our example of loading horses for transport, tools such as whips, wands, ropes, or other hand-held devices are commonly used to apply the R-. While these tools can function as subtle guiding aids, they also carry inherent risks. Pressure can escalate rapidly from a mild cue to fear-inducing stimulation or even P+, whether intentional or not. For horses with a history of these objects being used aversively, conditioned fear responses and conflict behaviors may be difficult to avoid. If a horse reacts suddenly, incidental contact with the object—due to either the horse’s movement or the handler’s reflexive response—may result in unintended P+. For these reasons, discerning R+ trainers are reluctant to advocate the inclusion of R-. Nonetheless, several trainers in our sample showed reasonably good progress toward loading using both R+ and R-. Interestingly, McLean has noted that trainers who identify as using fully R+ may include R- without recognizing or explicitly acknowledging it [13]; McLean, personal communication, 2026. From an educational perspective, it is therefore problematic when R- is misrepresented as R+.

Several demonstrations in our sample employed target training, in which a pre-conditioned positive stimulus (a target) is used to guide or entice (lure) the desired movement or response. As with other methods, skill is required to effectively lure horses in fear-eliciting contexts such as trailer loading. If not carefully managed, a delay of reinforcement may function as P-h, potentially intensifying approach–avoidance conflict responses and increasing frustration. This risk is especially pronounced in shaping procedures that involve repeated withholding of reinforcement for incremental progress. In our experience, targets, functioning as conditioned lures, along with intermittent or alternating primary and secondary R+, may be less likely to exacerbate conflict and frustration than primary appetitive lures.

Many horse trainers and other equine professionals have stressed the importance of using humane methods that support horse welfare in all aspects of domestic care and management [15]. In this work, we focused on trailer loading as an example of a common application of R+ training with horses. Our results highlight a gap between what trainers claim to be doing and what they may demonstrate. This is not a novel finding, as others have reported that equestrian knowledge of learning principles and equine behavior are often deficient [16,17,18,19,20]. As one example, Warren-Smith and McGreevy [20] found that only 11.9% of equestrian coaches correctly explained R-, and 38% correctly defined R+. Authors have pointed out that poor training advice often leads to compromised animal welfare [21]. When online demonstrations misrepresenting R+ and other forms of operant conditioning garner viewers and followers, they can distort public perception of both equine behavior and ethical training. 

With increasing public concern for the welfare of horses used in sport, interest in improved training methods is growing among horse professionals and other equestrian stakeholders. Equine welfare organizations are working towards fostering that interest by improving and disseminating educational materials regarding learning science. As an example, the International Society of Equitation Science (ISES) has a well-established history of providing scientifically sound educational materials on operant conditioning specific to horse training. Their influence in both print and online media has been effective in circulating accurate information in a user-friendly way to the horse-owning public (https://www.equitationscience.com (accessed on 26 May 2026)). 

The equestrian industry functions primarily on a social license to operate [22,23]. There are currently no legal or formal education requirements for an individual to operate as a horse professional in the capacity of animal trainer, riding instructor, coach, competitive judge, or other service provider outside of veterinary medicine. Some equestrian organizations offer certification programs in a specific area of expertise, such as the United States Pony Club, the Certified Horsemanship Association, and the US Eventing Association’s Certified Eventing Coaches program; however, these are all voluntary. One US state, Massachusetts, does require anyone operating as a riding instructor for hire to hold a state-issued license. Despite this requirement, the content on which applicants are tested does not include material relevant to learning theory or behavioral science [24]. Some legislative bodies have considered including educational standards for animal transport in general, and equestrian and animal welfare organizations in regions of the world with progressive animal welfare policies have discussed the possibility. Loading has been identified as one of the most stressful phases of the transport experience [25,26]. As the first step in the process, ensuring that the loading is minimally aversive for the horse is essential, and may help moderate subsequent stress once the vehicle is in motion. Whether through legislation or industry regulation, requiring handlers as well as drivers to be certified in humane and low-stress horse handling techniques would be beneficial to both horse welfare and human safety.

There are obvious limitations to consider. Perhaps the most notable, our observation of a positive association between the percentage of R+ trainer actions with positive horse responses in this sample, while it is consistent with other work, should be interpreted with caution. Selective editing of video demonstrations can misrepresent actual real-time events. Another potential concern is that these samples were evaluated by just one technician in consultation with the senior author. Results based on judgements of trainer actions and horse responses could vary among different observers. To mitigate influence of observer bias, both the trainer actions and the horse responses were operationally defined *a priori*, and recording was conducted in 15 s intervals with repeat replay as necessary to confirm each trainer action and corresponding horse response. It should also be noted that our sample of online video demonstrations was identified using only English language search terms. Therefore, it is unknown how well this sample represents online R+ educational materials worldwide.

## 5. Conclusions

Based on evaluation of this sample of online trailer-training video demonstrations, we conclude that a considerable proportion of online videos purporting to demonstrate R+ training of horses include R- and P+. To the extent that the equestrian community relies upon online video demonstrations to learn about R+ methods, these results are concerning in terms of the accuracy of information regarding operant conditioning.

The results of this study may help horse owners and the public to better understand the need to be selective when seeking horse training advice. They may also inspire further research into the application of R+, as well as the development of educational materials for the equestrian community. Attention to R+ training may help to shift industry norms away from aversive handling methods, leading to improved welfare for domestic horses used for sport, pleasure, or companionship.

## Figures and Tables

**Table 1:** Specific trainer actions common among various methods of training horses to load for transport.

POSITIVE REINFORCEMENT ACTIONS (R+)	
Trainer Action (TA)	Description
Click	Generate a ‘click’ or other standard sound to mark a desired behavior
Feed	Offer a food treat from the hand or feed pan, as a reward
Present Food	Offer the horse a food treat as incentive
Present Target	Place the target in the horse’s field of vision
Stand Idle	Stand still, quietly near the horse
Step in Trailer	Walk into the trailer, either fully or partially with the intent to have the horse follow
Visual Cue	Use hand as a visual guiding stimulus or target
Audible Cue	Utter vocal cue as conditioned positive prompt or conditioned reinforcer
Walk	Walk aside or ahead of the horse with the intention of leading the horse, with or without a halter and lead, and without applying pressure
**NEGATIVE REINFORCEMENT ACTIONS (R-)**	
Apply Spatial Pressure	Assert spatial dominance with body movements to displace the horse without physical contact
Apply Tactile Pressure	Touch the horse anywhere on his body with pressure
Pressure on Lead	Pull on the lead and/or halter
**POSITIVE PUNISHMENT ACTIONS (P+)**	
Apply Whip	Touch or strike the horse with the whip
Wave Hand Threateningly	Raise and wave arm and/or hand as a corrective measure
Wave Whip	Raise and wave the whip toward the horse as a corrective measure
**NEGATIVE PUNISHMENT ACTIONS (P-)**	
Block Horse’s Path	Stand in front of, behind, or aside of the horse, blocking desired movement
**OTHER TRAINER ACTIONS**	
Adjust Trailer Component	Adjust the trailer ramp, door, divider, or other component
Vocalize	Make audible sounds that are not intended as cues

**Table 2:** Horse behavioral responses.

POSITIVE BEHAVIORS	
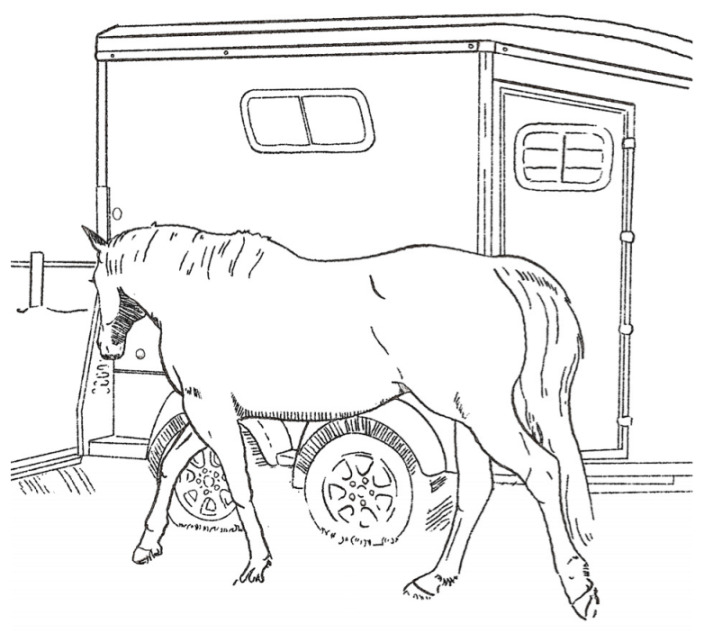	**Approach** Approach the trailer voluntarily
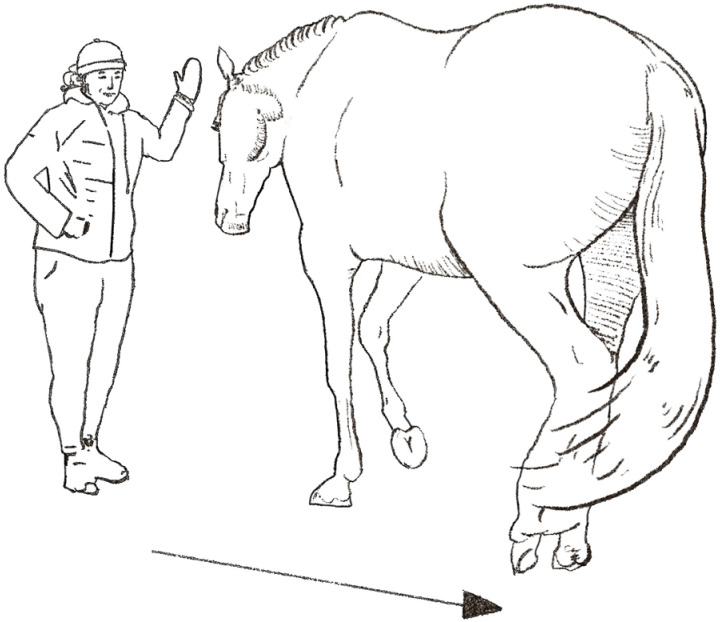	**Back Up When Directed** Walk backward in response to a cue from trainer
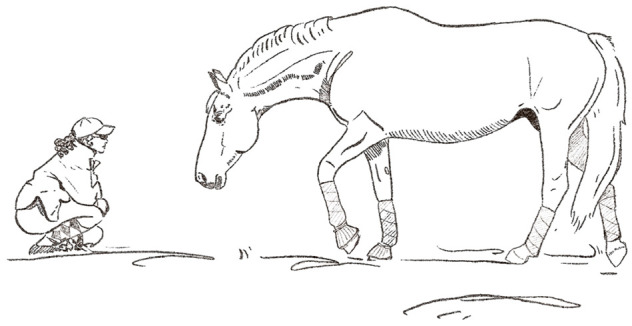	**Engage with Trainer** Sniff, nuzzle, investigate, or otherwise interact with trainer
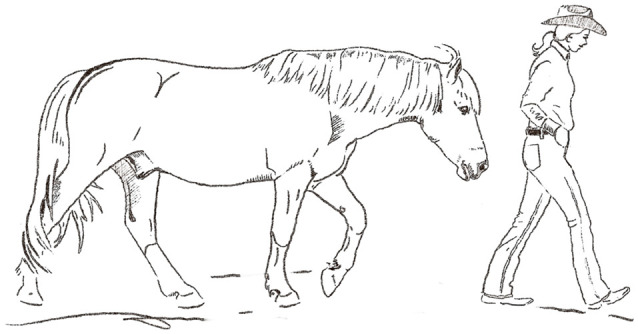	**Follow Trainer** Walk close behind trainer at a similar pace
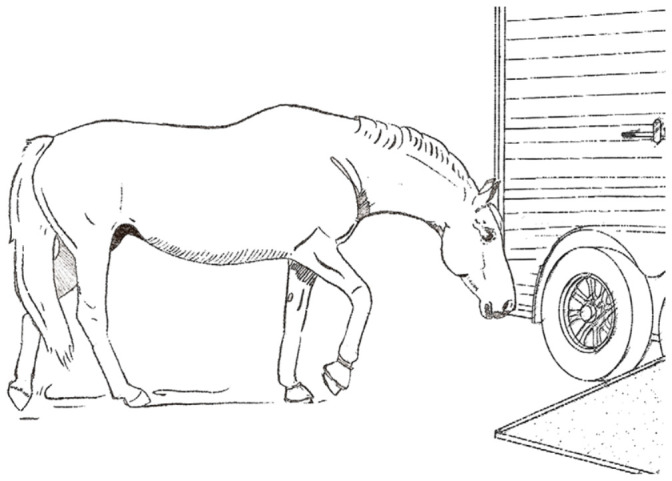	**Investigate Trailer** Sniff, touch, or nuzzle any portion of the trailer
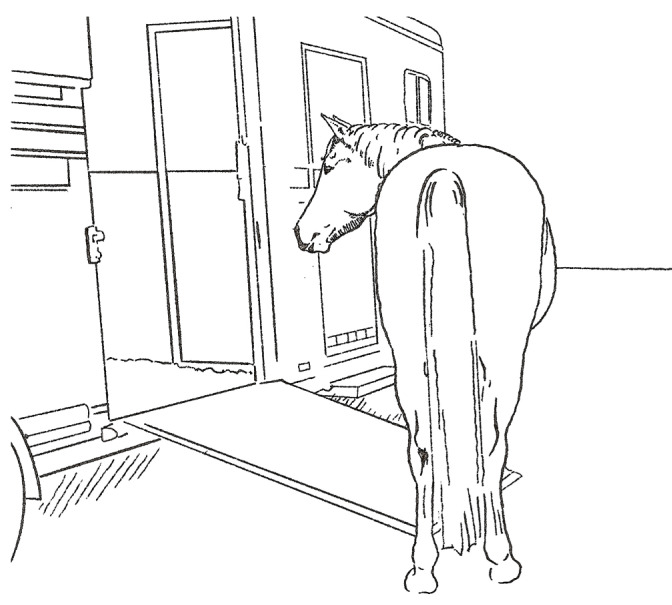	**Look in with Interest** Stand at entry of the trailer, looking into the interior with apparent interest
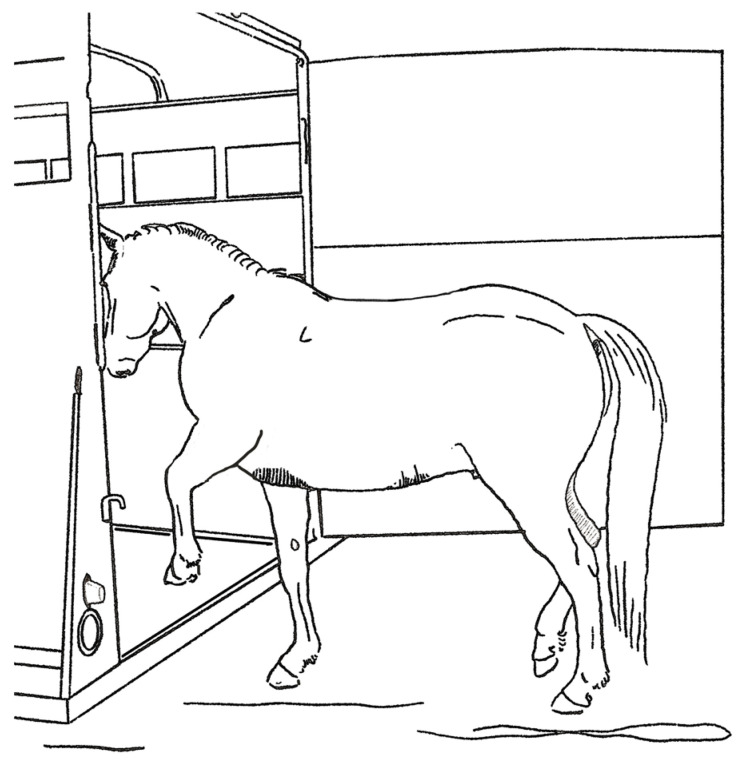	**One Foot On** Place one fore foot on the ramp or upon the step
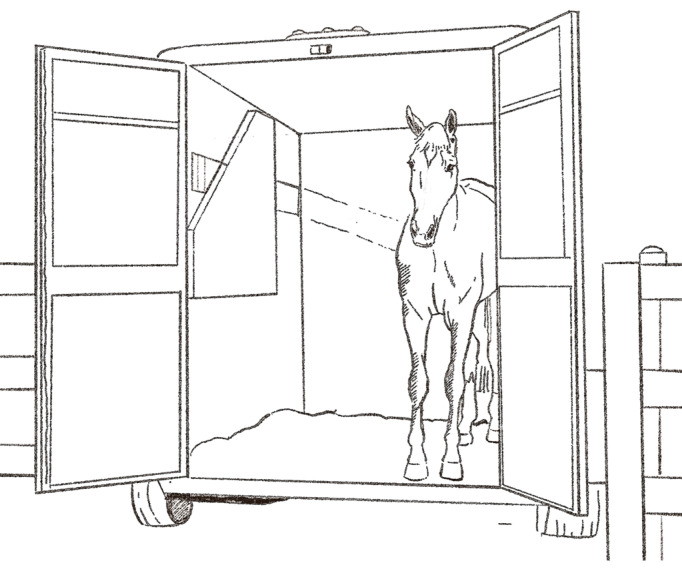	**Stand In Trailer** Enter and stand inside the trailer
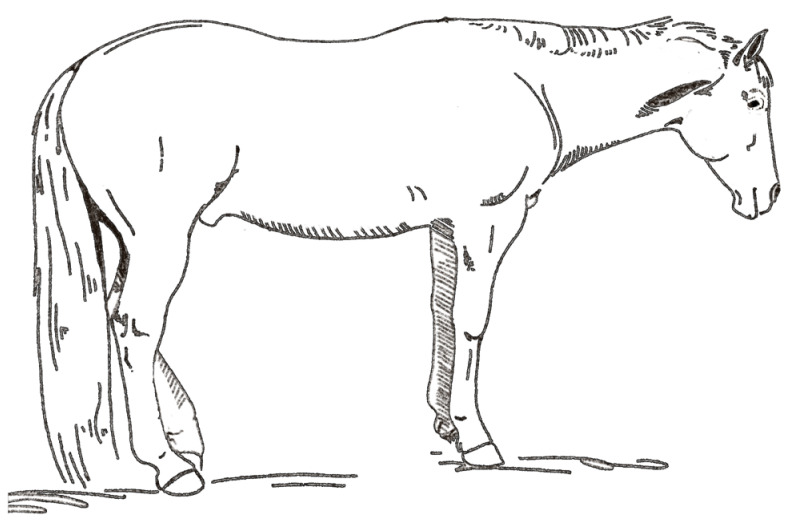	**Stand Relaxed** Stand with ears rotated laterally, head at or slightly above the withers, tail hanging still and resting against perineum
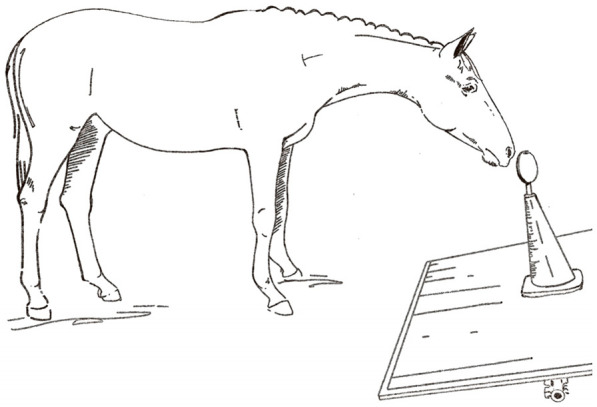	**Touch Training Target** Touch a stationary or hand-held training target with muzzle
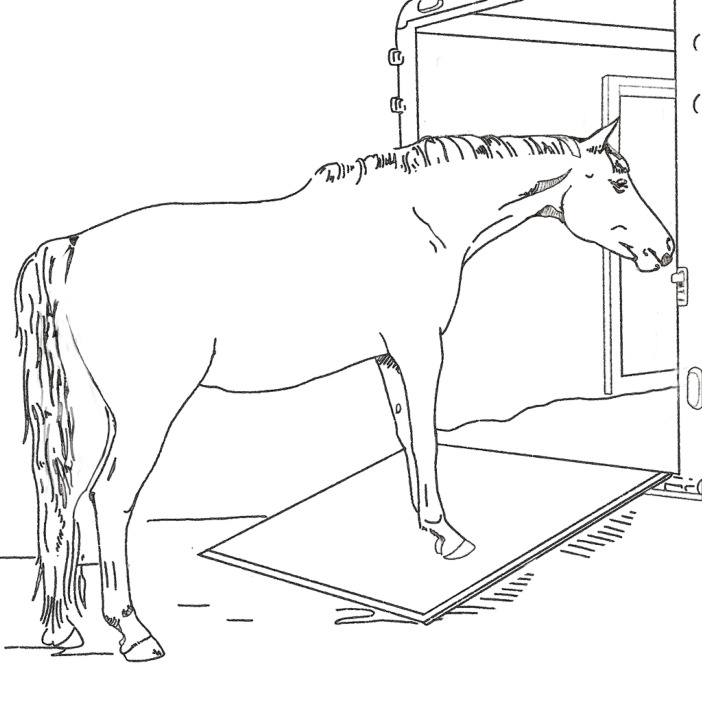	**Two Feet On** Place two front feet on the trailer ramp or step, persistently, or for more than 5 s
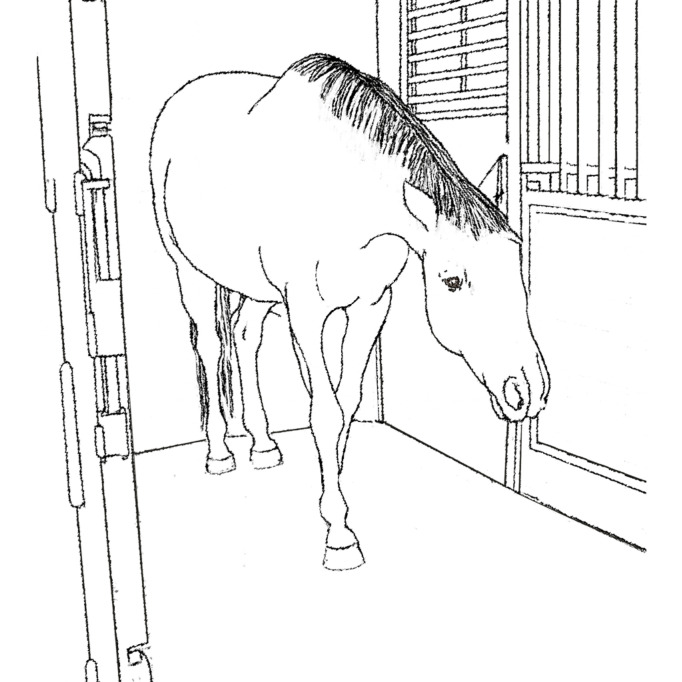	**Walk In** Walk fully into the trailer
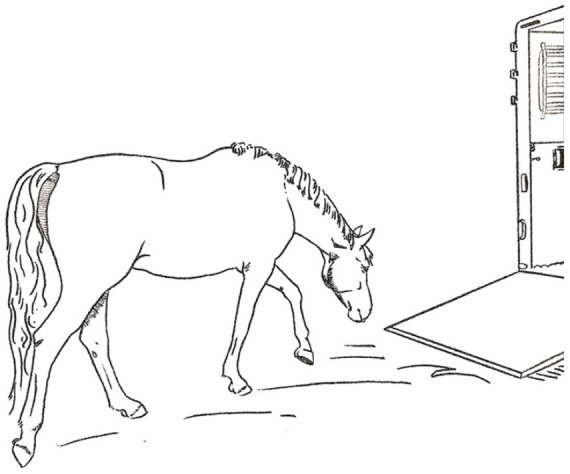	**Walk Toward Trailer** Walk toward trailer as if intending to enter
**NEUTRAL BEHAVIORS**	
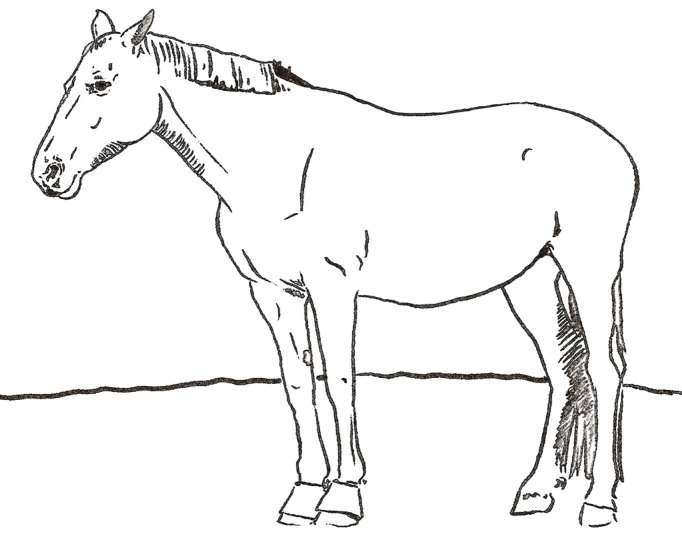	**Non-Responsive** Standing, non-responsive, unengaged
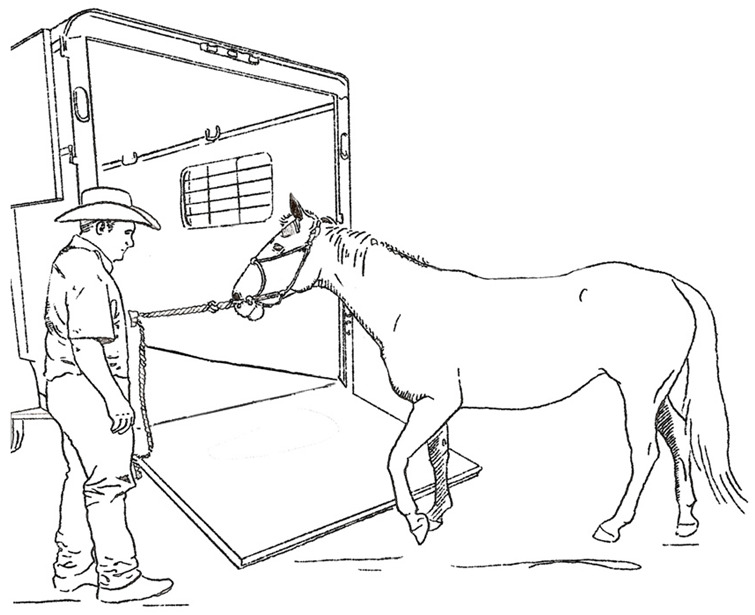	**Yield to Pressure** Move in response to pressure
**NEGATIVE BEHAVIORS**	
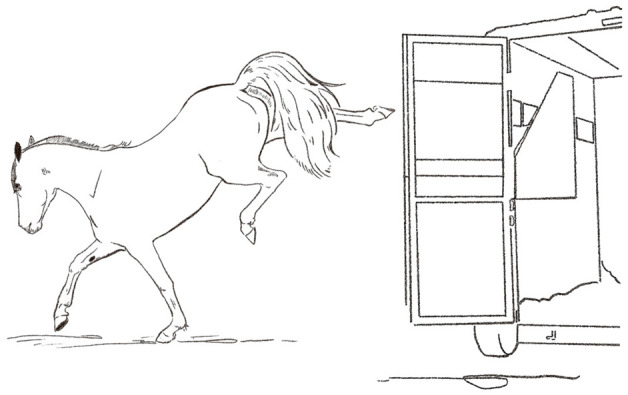	**Buck** Lowering of the head and neck and with weight shifted to the forelegs, both hind legs lift off the ground with simultaneous backward extension
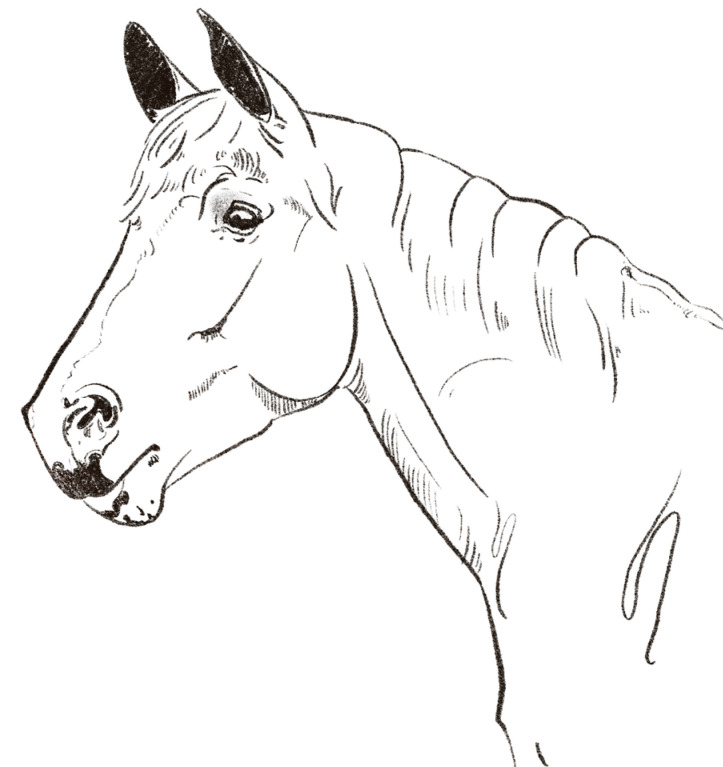	**Concerned Facial Expression** Sclera visible and/or inner brow raised, tense facial muscles
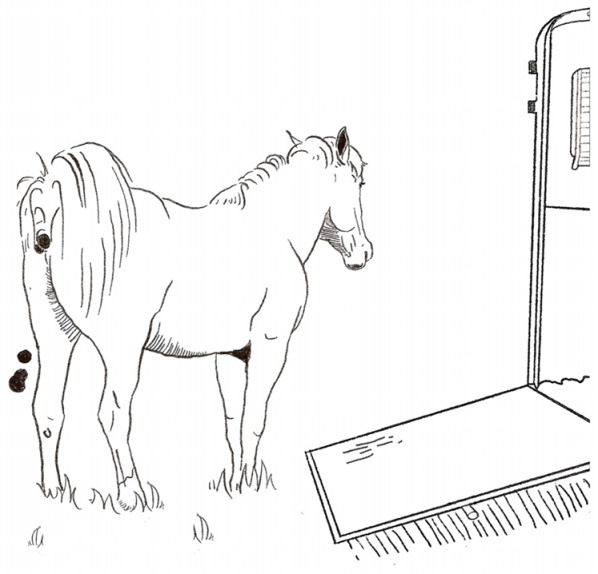	**Defecate** Pass feces
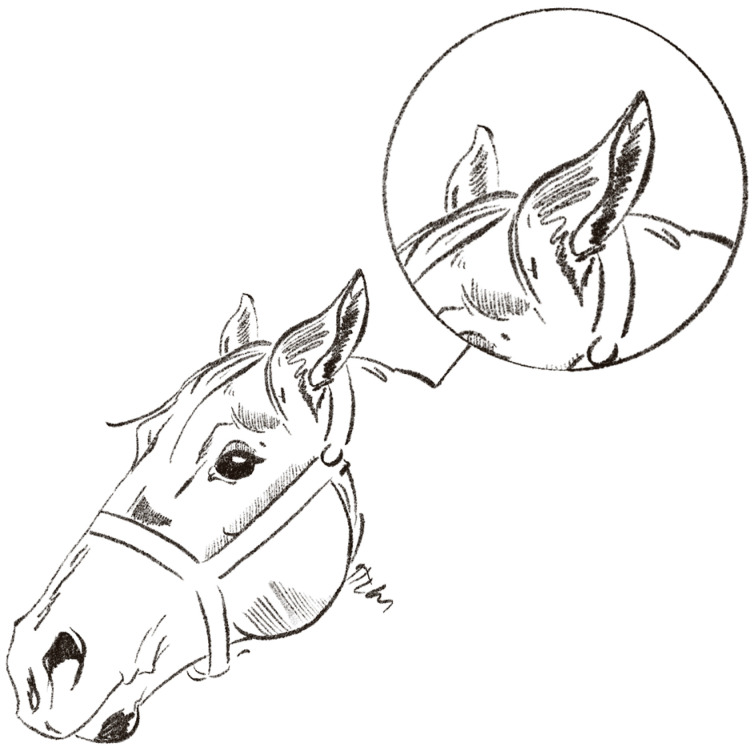	**Ears Back** Both ears rotated backward
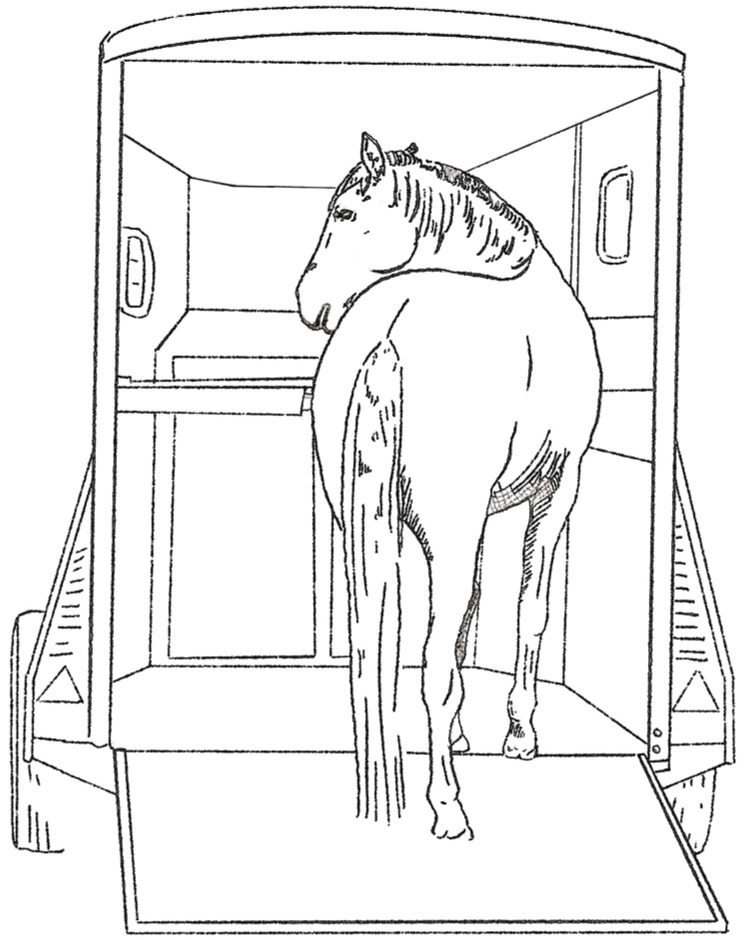	**Look Back** While oriented toward the trailer, look back with expression of concern
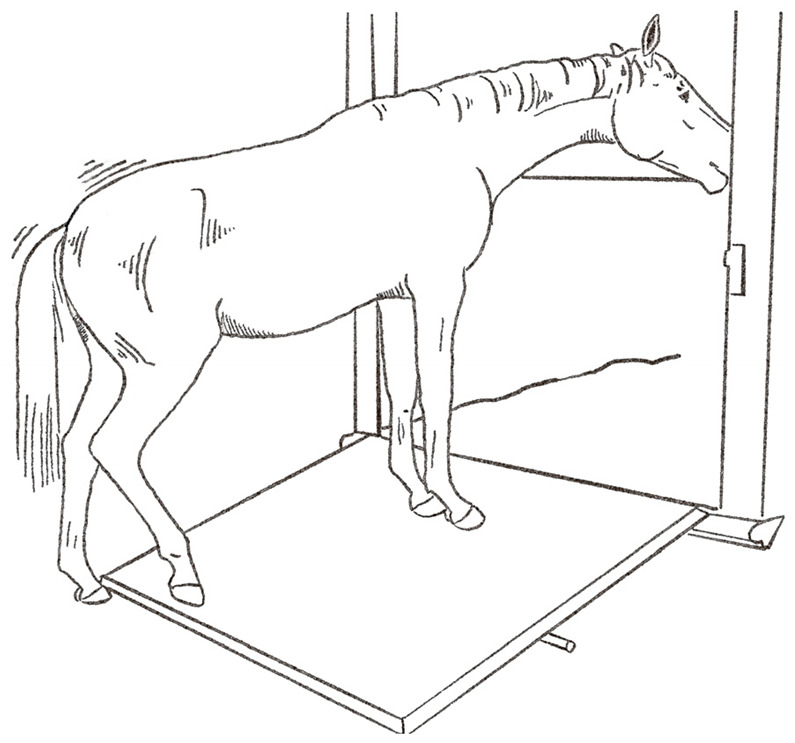	**Muscle Trembling** Twitching or trembling of muscles without locomotion
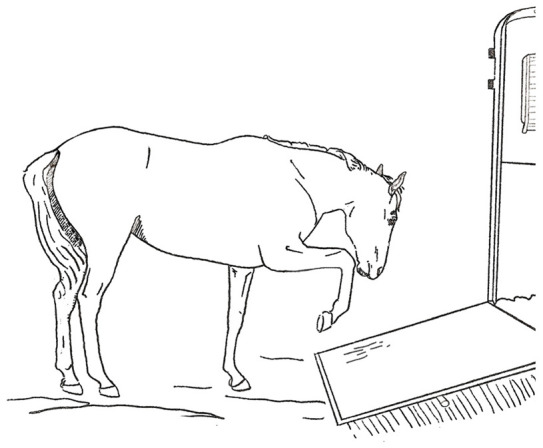	**Paw** (Anxious/Frustration Form) Reach a forelimb cranially and drag the hoof along or above the substrate, while sweeping caudally, often in rhythmic series
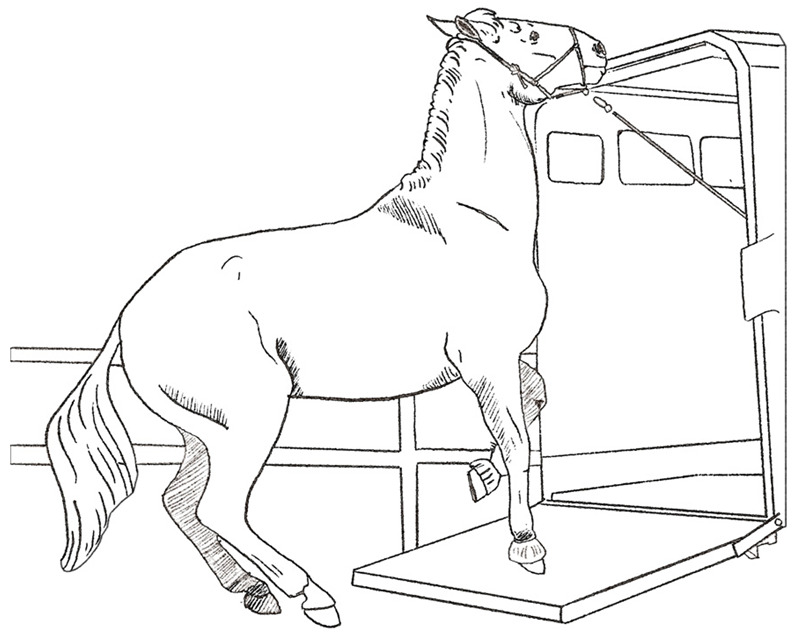	**Pull Back** With head elevated, pull back on lead rope away from trailer
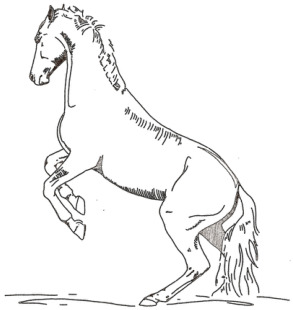	**Rear** Raise forelegs into the air, supporting the body on the hind legs, resulting in a near-vertical position
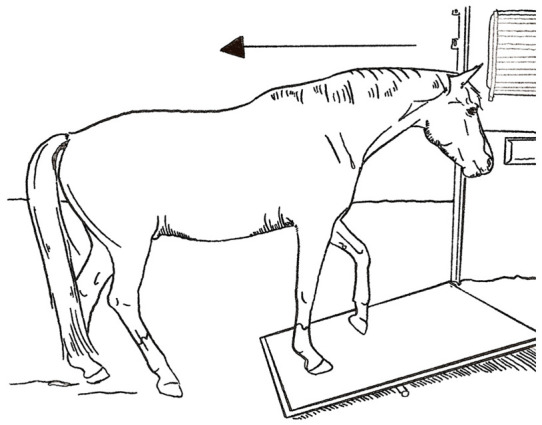	**Retreat Backward** Back away from the trailer
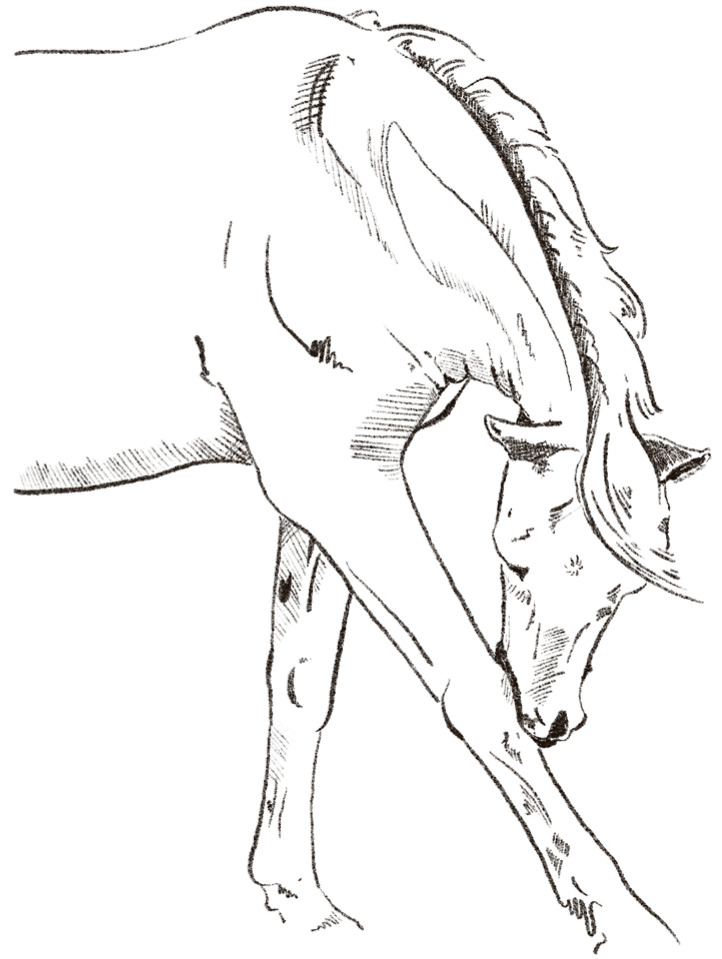	**Rub Muzzle** Rub muzzle on a foreleg
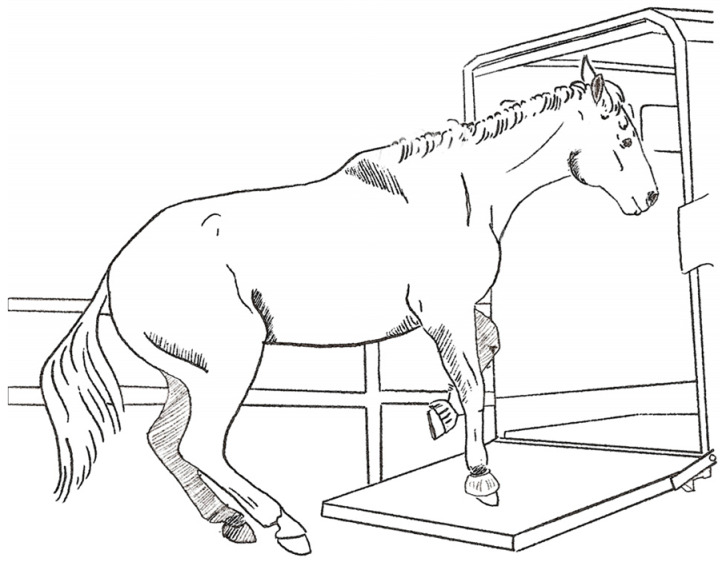	**Scramble Back** Quickly move backward in a disorganized and anxious manner
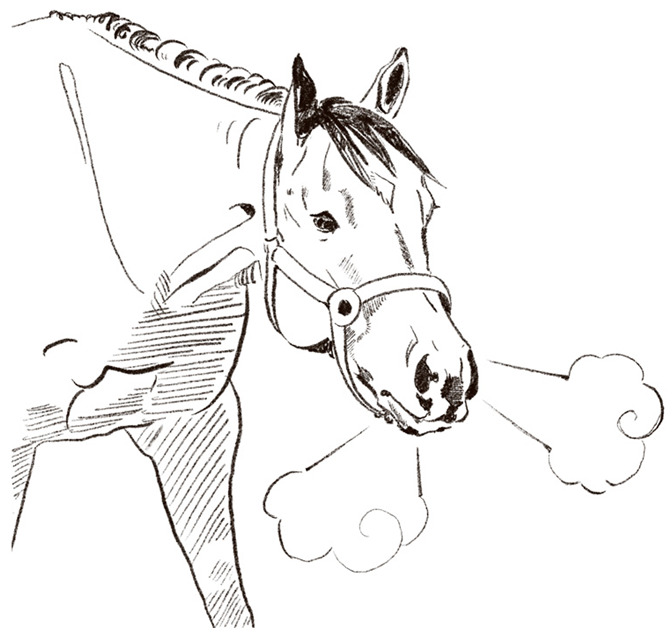	**Snort** Forceful, quick exhalation through the nose
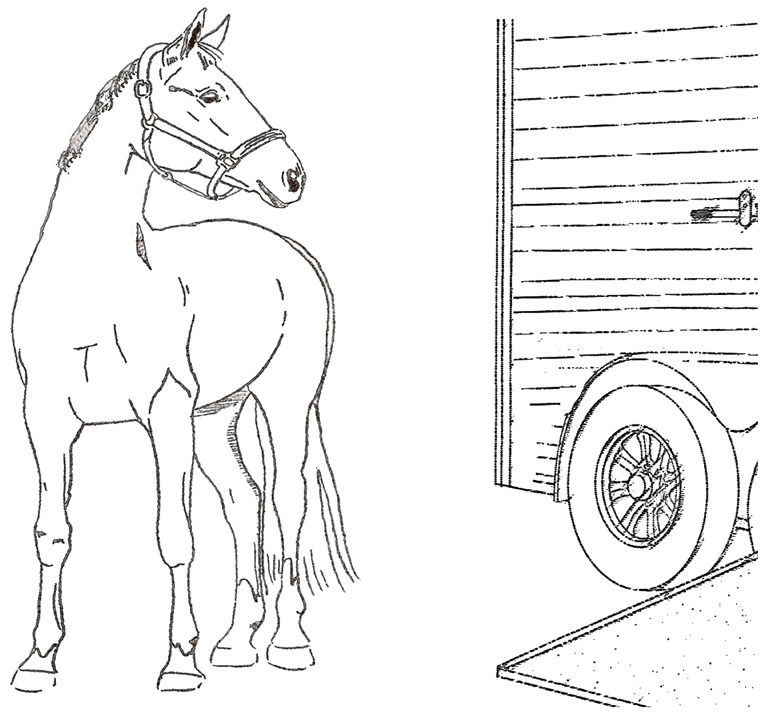	**Stand Alert** Stand alert with head elevated above the withers, ears directed toward perceived threat, motionless, tightened muzzle, concerned facial expression
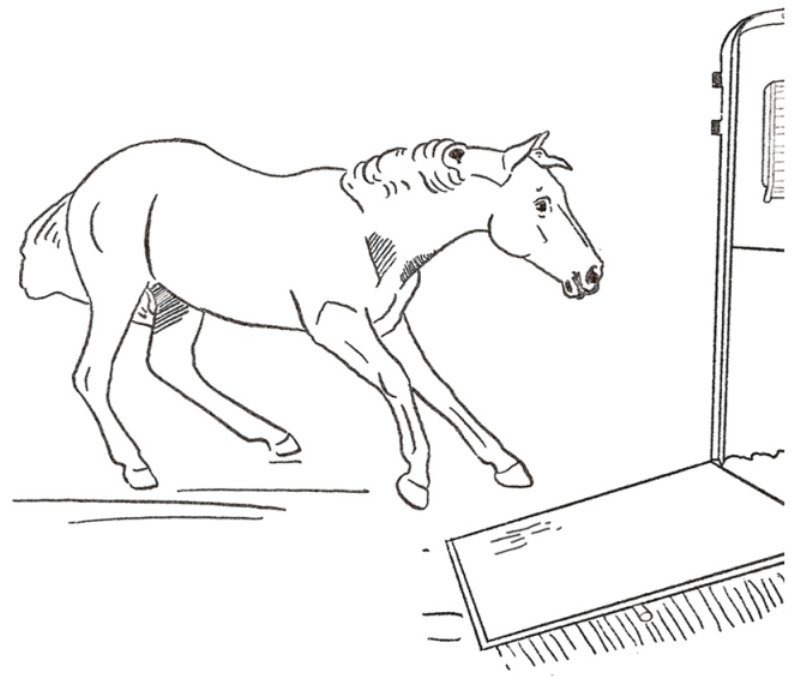	**Startle** Quick movement in response to stimuli
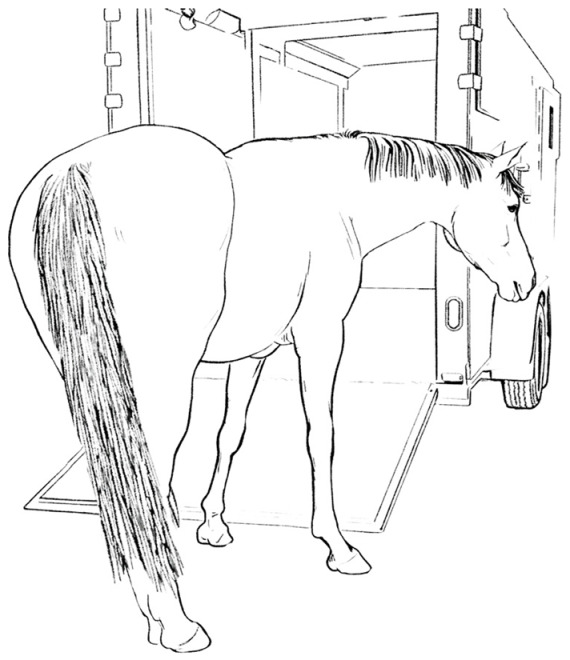	**Step to the Side** Step to the left or right of the trailer entry
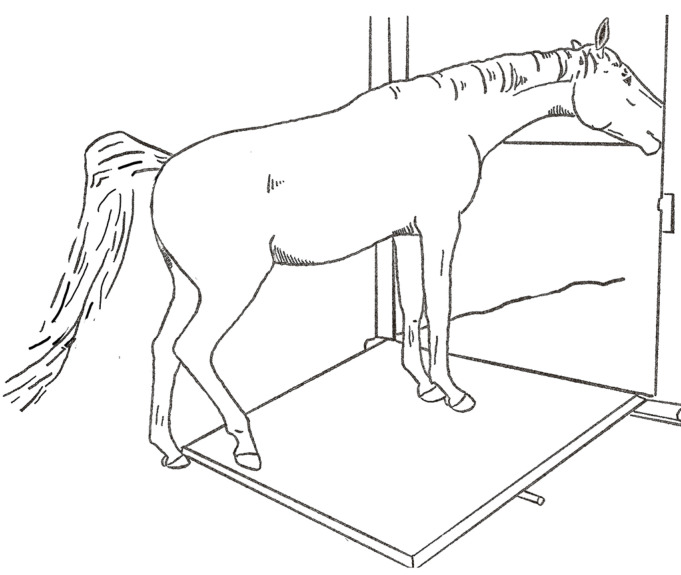	**Tail Raised** Tail held off the perineum
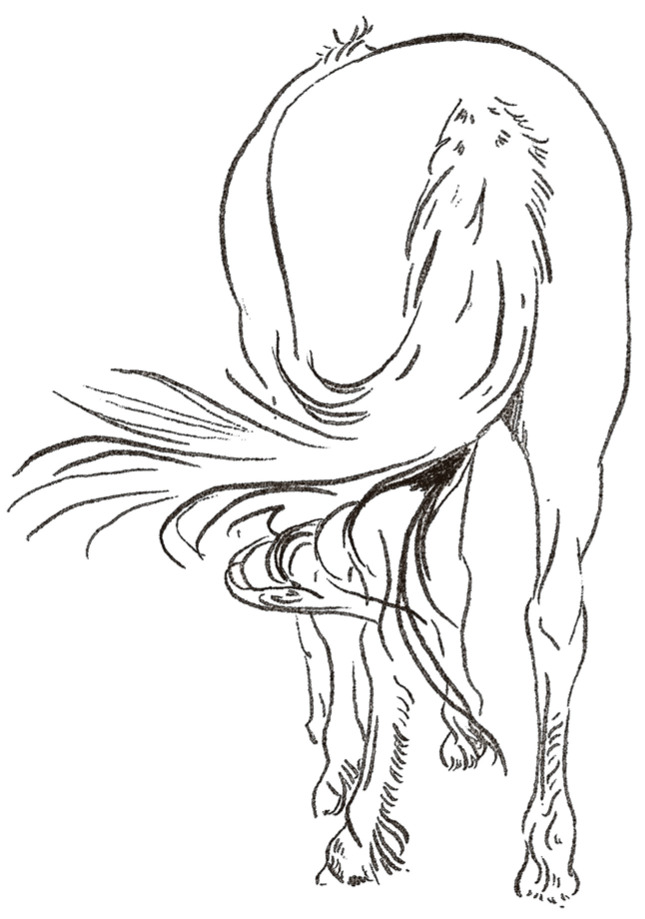	**Tail Swish** Tail moves quickly at least once
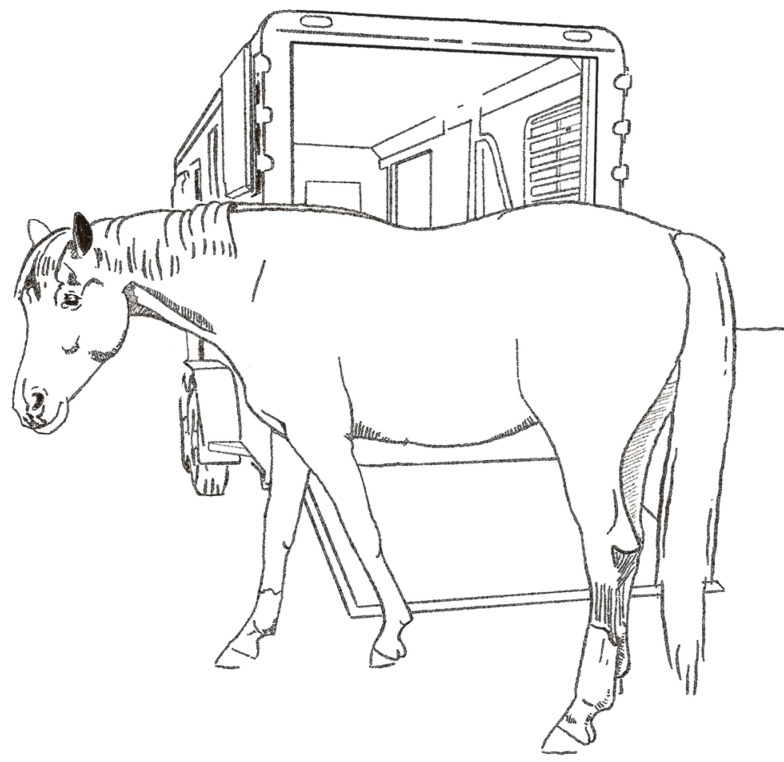	**Turn Away** Turn away from the trailer
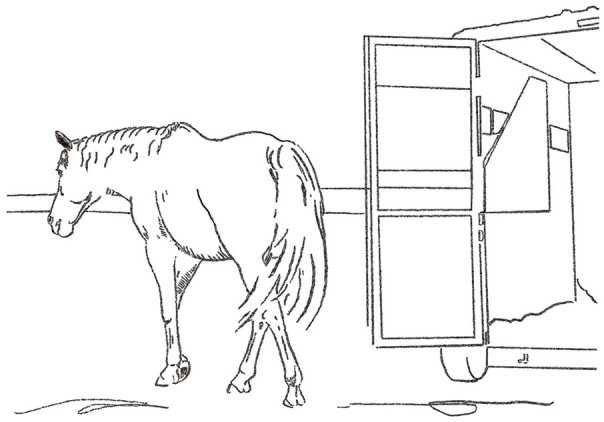	**Walk Away** Walk away from the trailer
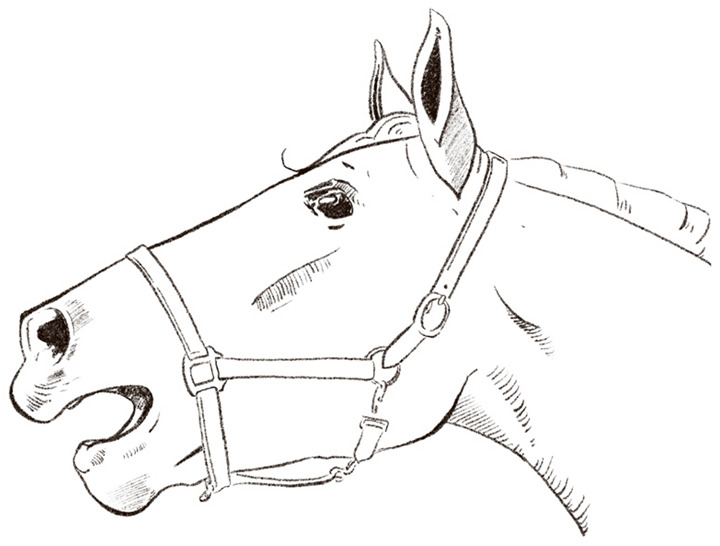	**Whinny** Emit a high-pitched stress vocalization

## Data Availability

The raw data supporting the conclusions of this article will be made available by the authors upon request.
